# Perceptual task induces saccadic adaptation by target selection

**DOI:** 10.3389/fnhum.2015.00566

**Published:** 2015-10-20

**Authors:** Alexander C. Schütz, David Souto

**Affiliations:** ^1^Abteilung Allgemeine Psychologie, Justus-Liebig-Universität GießenGießen, Germany; ^2^Allgemeine und Biologische Psychologie, Philipps-Universität MarburgMarburg, Germany; ^3^Department of Neuroscience, Psychology and Behaviour, University of LeicesterLeicester, UK

**Keywords:** saccadic adaptation, attention, visual perception, reinforcement learning, target selection

## Abstract

Adaptation of saccades can be induced by different error signals, such as retinal position errors, prediction errors, or reinforcement learning. Recently, we showed that a shift in the spatial goal of a perceptual task can induce saccadic adaptation, in the absence of a bottom-up position error. Here, we investigated whether this top-down effect is mediated by the visibility of the task-relevant object, by reinforcement due to the feedback about the perceptual judgment or by a target selection mechanism. Participants were asked to discriminate visual stimuli arranged in a vertical compound. To induce adaptation, the discrimination target was presented at eccentric locations in the compound. In the first experiment, we compared adaptation with an easy and difficult discrimination. In the second experiment, we compared adaptation when feedback about the perceptual task was valid and when feedback was provided but was unrelated to performance. In the third experiment, we compared adaptation with instructions to fixate one of the elements in the compound—target selection—to the perceptual task condition—target selection and discrimination. To control for a bottom-up stimulus effect, we ran a fourth experiment in which the only instruction was to look at the compound. The saccade amplitude data were fitted by a two-state model distinguishing between an immediate and a gradual error correction process. We replicated our finding that a perceptual task can drive adaptation of saccades. Adaptation showed no effect of feedback reliability, nor an effect of the perceptual task beyond target selection. Adaptation was induced by a top-down signal since it was absent when there was no target selection instruction and no perceptual task. The immediate error correction was larger for the difficult than for the easy condition, suggesting that task difficulty affects mainly voluntary saccade targeting. In addition, the repetition of experiments one week later increased the magnitude of the gradual error correction. The results dissociate two distinct components of adaptation: an immediate and a gradual error correction. We conclude that perceptual-task induced adaptation is most likely due to top-down target selection within a larger object.

## Introduction

Saccadic adaptation is a gradual change in visuomotor mapping that reduces errors in saccade amplitude or direction. This plasticity in the eye movement system is typically studied with the intra-saccadic step paradigm (McLaughlin, 1967), in which the eye movement target is shifted during the saccade (for reviews, see Pelisson et al., 2010; Herman et al., 2013). This shift of the eye movement target induces a post-saccadic visual error, which is corrected over the course of several saccades. A major focus of the study of saccadic adaptation is the identification of the error signals that drives adaptation. It has been shown that corrective saccades (Wallman and Fuchs, 1998; Bahcall and Kowler, 2000) and proprioceptive signals (Lewis et al., 2001) are not necessary to induce adaptation. Instead retinal position and prediction errors contribute predominantly to adaptation (Bahcall and Kowler, 2000; Wong and Shelhamer, 2011; Collins and Wallman, 2012).

Recently, we showed that a perceptual task can induce saccadic adaptation, even in the absence of a bottom-up visual error (Schütz et al., 2014). In this paradigm, observers have to discriminate a character that appears at different locations in a peripherally appearing compound stimulus. Over time the eye movements land closer to the perceptually relevant character in the compound. Several properties of this effect are reminiscent of classical saccadic adaptation: First, saccade amplitudes change gradually over time and aftereffects are present after the adaptation phase. Second, the magnitude of changes and the rate of change are similar to an intra-saccadic step paradigm. Third, changes in saccade direction lead to changes in the curvature of saccades as with an intra-saccadic step paradigm (Chen-Harris et al., 2008). Fourth, the effects partially transfer to reactive saccades without a perceptual task. Due to these similarities, we called the effect saccadic adaptation. We also showed in our previous study that adaptation does not take place when observers see the same stimuli, but are only instructed to look at the peripheral compound without engaging in the perceptual task. Hence, adaptation induced by the perceptual task must originate in a top-down error signal, not stimulus saliency. Since the task-relevant element changed location during adaptation, the top-down signal driving adaptation necessarily contains a target selection component. However, we hypothesized that the perceptual-task induced adaptation could be mediated or modulated by other factors on top of target-selection.

First, top-down adaptation could be mediated by visual uncertainty about the properties of the task-relevant object. In a visual search paradigm, observers’ eye movement patterns are similar to an ideal observer that has full knowledge of its sensitivity distribution across the visual field and that uses this knowledge to maximize the information gain with each eye movement (Najemnik and Geisler, 2005; Geisler et al., 2006). These findings can be transferred to our perceptual-task adaptation paradigm, especially since the discrimination target is only presented briefly after the saccade, allowing no refixation. Since there are several distractors and only one task-relevant element in the peripheral compound the information gained by the initial saccade would be maximal if it lands on the task-relevant element and would decrease with increasing distance to the task-relevant element. In this case, we expect faster and stronger adaptation with stimuli that are more difficult to discriminate and for which there is a larger discrimination benefit for being on target.

Second, the teaching signal could come from an external source—i.e., a beep indicating an incorrect response—instead of an internal source—improved visibility. The feedback about the perceptual judgment could then act as reinforcement signal. In that sense a positive feedback would tell the eye movement system that the saccade was useful and that it should keep the visuomotor mapping. A negative feedback would tell the eye movement system that the saccade was inadequate and that the eye movement metrics have to be adjusted. Although the feedback does not directly specify in which direction the movement has to be corrected, saccade adaptation induced by reinforcement has been recently demonstrated (Madelain et al., 2011). A prediction based on the effect of reinforcement would be that adaptation without feedback or uninformative feedback should be weaker or absent.

Third, top-down adaptation could merely depend on the selection of the task-relevant element as goal for the eye movement. In this case, the error between the landing position of the eye and the location of the task-relevant element would serve as error signal and the consequences of the error for the perceptual task would be ignored. In contrast to the traditional view of saccadic adaptation this error could depend on the top-down target selection of the visual stimulus, which in turn depends on the requirements of the perceptual task. This interpretation would be consistent with findings that adaptation is specific to the eye movement target and not affected by intra-saccadic position changes of the background or distractors (Madelain et al., 2010, 2013). Moreover, saccade adaptation induced by target selection might not even require eye movements to take place, since adaptation of covert spatial attention shifts transfers to eye movements (McFadden et al., 2002).

In this study, we tested the three aforementioned hypotheses about the perceptual-task induced saccadic adaptation effects by manipulating the visual uncertainty of the task-relevant object, by manipulating the validity of the feedback about the perceptual judgment, and by comparing adaptation in the perceptual task condition to simple target selection. We distinguished different effects of training and visual uncertainty by fitting a model, in which adaptation results from an immediate and a gradual error correction process. As a control condition for bottom-up effects we ran an experiment with the same stimuli but without any instruction to target or discriminate one of the elements in the compound.

## Materials and Methods

### Observers

Thirty-eight students from Giessen University (31 female, average age 24 ± 6 years) participated in these experiments. Experiments complied with the principles of the Declaration of Helsinki and were approved by the local ethics committee LEK FB06 at Giessen University (Proposal Number 2013-0020). We had to exclude the data of six other observers because more than 30% of trials in the adaptation conditions were invalid (cf. eye movement recording). Observers participated in these experiments for partial course credit and received monetary rewards depending on their performance in the perceptual task in Experiments 1 and 2. Observers were instructed that the total number of correct judgments will be converted into monetary rewards. They received between 8 and 12aaa. Valid data sets were obtained from 10, 10, 9, and 9 observers, in Experiments 1 to 4, respectively.

### Experiments

In three experiments, we tested if the effects of the perceptual task are modulated by task difficulty (easy or difficult), by the feedback about the perceptual judgment (valid or random) or by mere target selection. An additional control experiment tested whether adaptation effects could be driven by bottom-up stimulus salience alone. In Experiment 1 (*n* = 10), we compared an easy (gap size: 100% of square side) and a difficult (gap size: 20%) perceptual task. Observers did not receive any feedback about their perceptual judgments. The order of easy and difficult conditions was counterbalanced across subjects. In Experiment 2 (*n* = 10), we compared a valid feedback condition with a random feedback condition. Observers heard a beep if their perceptual judgment was incorrect. The valid condition was recorded first, to measure the average proportion of correct responses for each observer. The random condition was recorded afterward; here feedback was given randomly, but at the same rate as in the valid condition. In Experiment 3 (*n* = 9), we compared a difficult perceptual task condition to a condition in which observers were merely instructed to fixate the same black square that was the target in the perceptual task. The order of these conditions was counterbalanced across subjects. The stimuli were the same as in the difficult condition of Experiment 1. Experiment 4 (*n* = 9) was a control for testing whether bottom-up visual salience of the target within the compound could generate saccade adaptation. Observers saw the same stimuli as in Experiment 3, but were instructed to saccade to the peripheral compound, ignoring element colors. In all experiments, adaptation conditions were separated by at least 1 week.

### Adaptation Procedure

**FIGURE 1:**
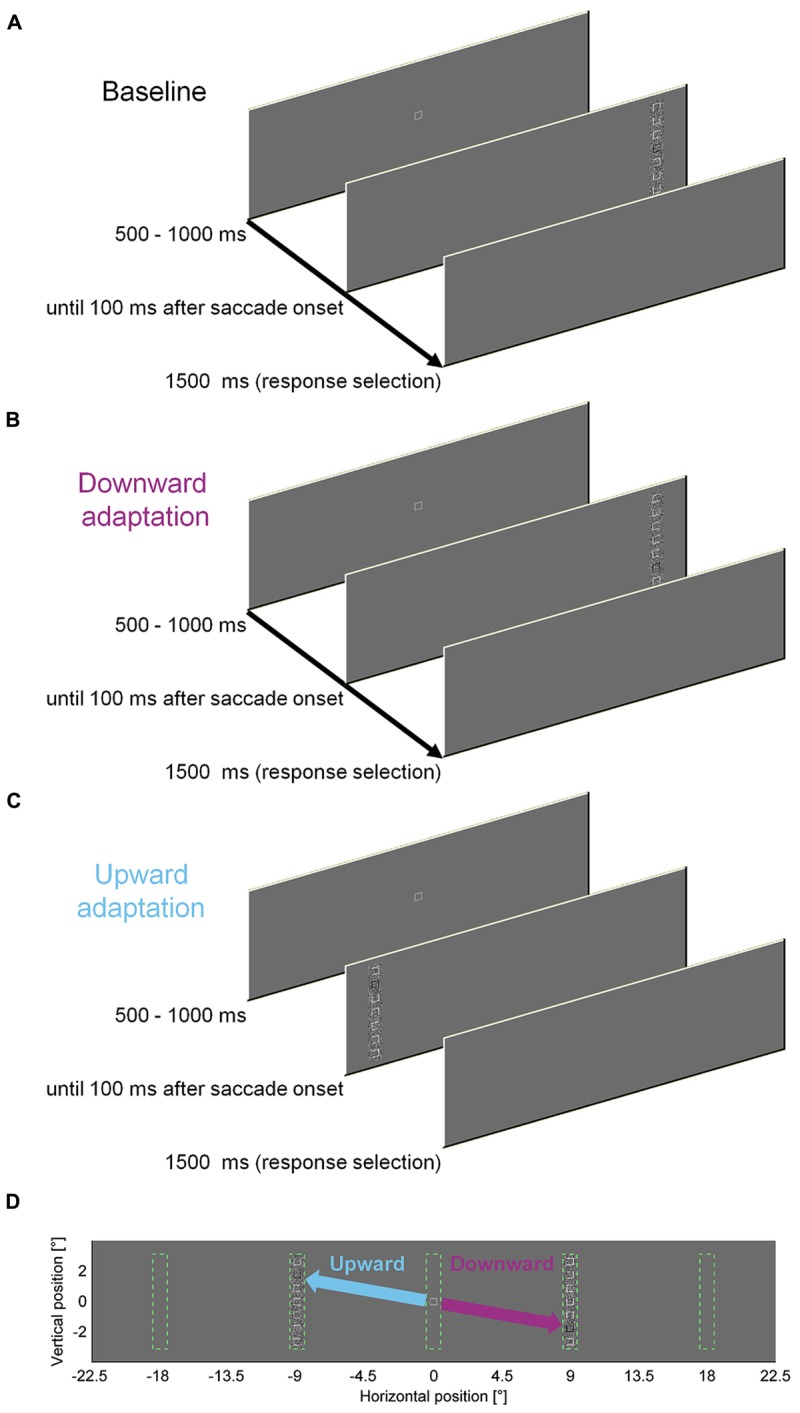
**Experimental paradigm to induce saccadic adaptation by a perceptual task.** **(A)** Time course of one pre- or post-adaptation trial. In 100 pre- and 300 post-adaptation trials, the task-relevant square was located at the center of the compound. **(B)** Time course of one downward adaptation trial. In 300 adaptation trials, the task-relevant square was either located at the top or at the bottom of the compound, depending on the direction of the horizontal step. **(C)** Time course of one upward adaptation trial. **(D)** Potential locations of the compound and adaptation manipulation. Vertical compounds of squares could appear 9° to the left or to the right of initial fixation. Upward steps for leftward saccades and downward steps for leftward saccades were counterbalanced across observers. Compared to a previous study (Schütz et al., 2014), the individual elements were squares rather than characters. Moreover the task-relevant element was marked in black throughout the stimulus presentation, as in Experiment 8 in Schütz et al. (2014).

To measure the influence of a perceptual task on saccade amplitudes, we asked observers to discriminate the location of a gap in a square that was presented within a vertical compound of seven squares. We designed the discrimination task to require foveal acuity, such that observers had to saccade to the compound to solve the task. To induce saccadic adaptation, the discrimination square was presented at different locations within the peripheral compound in different blocks of trials (**Figure 1**). In our previous study, letters were used and the target stimulus was blackened after the saccade in most experiments. However, keeping it blackened throughout the presentation as in the present experiments did not affect adaptation rate or perceptual performance (Experiment 8 in Schütz et al., 2014). In the first 100 (pre-adaptation) and last 300 (post-adaptation) trials the discrimination square was located at the central location (position four) in the compound. In the 300 (adaptation) trials in-between, the discrimination square was located at eccentric locations. For half of the observers it was located two squares down from the center for rightward saccades and two squares up for leftward saccades. For the other half of the observers this association was reversed. Consequently, the saccade direction had to be rotated upward or downward, depending of the step direction. The adaptation procedure was similar to the cross-axis adaptation in our previous study (Schütz et al., 2014). We used cross-axis adaptation (Deubel, 1987; Chen-Harris et al., 2008; Schütz and Souto, 2011), because the data are expected to be cleaner since constant errors and variability of saccade endpoints are larger in the movement direction than orthogonal to it (van Opstal and van Gisbergen, 1989; van Beers, 2007).

### Visual Stimuli

Discrimination targets were squares with a side length of 0.38° (degrees of visual angle) and a gap on one side. Line width was 1 pixel or 0.03°. The squares were displayed in black or white in front of a random noise background extending 0.2° beyond the squares. The contrast of the squares was 0.6 and the noise contrast was 0.8. Seven squares were aligned vertically for a total height of 5.5°. A single square was used as fixation target. In Experiment 1, the gap was 100% of one side in the easy condition and 20% in the difficult condition. In Experiment 2, the gap was 20% in all conditions.

### Experimental Procedure

Observers had to fixate a fixation square. After 500–1000 ms, the fixation square disappeared and the stimulus compound appeared at an eccentricity of 9°, randomly to the right or to the left, except when the fixation target reached the farthest position to the left or to the right. On the next trial, a new fixation square appeared at the previous center of the compound, which then performed a random-walk across five potential positions on the monitor during the experiment (**Figure 1**). One of the squares outline was black while the others were white, indentifying it as the discrimination target. The compound was displayed for 100 ms after saccade onset. The observers had 1500 ms after saccade onset to indicate the location of the gap by a key press. In Experiment 1 a beep only was played if they did not provide an answer. In Experiment 2, in the valid feedback condition, the same beep was also played if their perceptual judgment was incorrect. In the random condition, the error feedback was not related to the perceptual judgment and an error was reported randomly at the same rate as the subject made errors in the valid condition. Actual detection rates in valid and random feedback conditions were not significantly different [*t*(9) = 0.43, *P* = 0.678] and were highly correlated [*r*(9) = 0.78, *P* = 0.008]. In the target selection condition of Experiment 3 and in Experiment 4, observers did not have to engage in the perceptual task.

Breaks of 30 s occurred every 100 trials and observers were advised to close their eyes during those breaks. In Experiments 1 to 3, observers were also instructed that the location of the discrimination square remained identical within each block of 100 trials.

### Fixation Baseline

In a fixation baseline measurement (*n* = 29), we assessed the effect of gap size and eccentricity on perceptual performance. Observers had to fixate a fixation square at the center of the screen. After 500–1000 ms, the compound stimulus was presented for 100 ms at the center of the screen. The relevant black square was located at positions 2, 3, 4, 5, and 6 from the top. The gap size was either 20 or 100%. Each combination of gap size and position was presented 20 times, and the positions were blocked within the experiment, such that the task relevant position was completely predictable. Observers received valid feedback about their perceptual judgment.

### Materials

Stimuli were displayed on a 23.6-inch VIEWPixx monitor (VPixx Technologies Inc., Saint-Bruno, QC, Canada) driven by a AMD FirePro V4900 graphics board with a refresh rate of 120 Hz. At a viewing distance of 48.5 cm, the active screen area subtended 61° horizontally and 34° vertically. With a spatial resolution of 1920 × 1080 pixels, this results in 32 pixels/°. The luminance of white, gray, and black pixels was 228, 30, and 0.6 cd/m^2^, respectively. Stimulus presentation was controlled by Matlab (MathWorks, Natick, MA, USA) using the Psychophysics toolbox (Brainard, 1997; Pelli, 1997). The experiments took place in a dark room and the observer’s head was stabilized by a forehead and chin rest.

### Eye Movement Recording

Eye position signals of the right eye were recorded with a video-based eye tracker (EyeLink 1000; SR Research, Kanata, ON, Canada) and were sampled at 1000 Hz. The eye tracker was driven by the Eyelink toolbox (Cornelissen et al., 2002). Saccade onsets were detected online when eye velocity of two subsequent samples exceeded 50 and 100 °/s, respectively. For oﬄine analysis, the Eyelink saccade parser was used, which uses a velocity and acceleration threshold of 22 °/s and 3,800 °/s^2^, respectively. We excluded on average 9.5% of trials, if no saccade onset was detected after 2 s (2.1%), if the horizontal saccade amplitude was smaller than 5° or larger than 13° (9.1%) and if the absolute vertical saccade amplitude exceed 3° (2.2%), corresponding to a saccade outside the stimulus compound.

### Data Analysis and Modeling

We used the average vertical saccade amplitude in the 50 pre-adaptation trials and in the last 50 adaptation trials to quantify the effects of adaptation on eye movements. The effects of experimental condition, adaptation phase and saccade direction were statistically tested by a repeated measures ANOVA, followed-up by paired *t*-tests. The mean and standard deviation across observers are reported as descriptive statistics. A statistical significance level of 0.05 was adopted.

To estimate the relative contributions of an immediate and a gradual adjustment of saccades, we extended a state-space model that has been used successfully to model classical saccadic adaptation (Srimal et al., 2008). In this extension, we added a second state, representing the immediate error correction. This modified model has been used previously to model on-axis and cross-axis adaptation (Schütz et al., 2014). The model assumes that the gain of a saccade *y_n_* on a given trial *n* is determined by a weighted combination of the state of an immediately adapting process *z_i_* and the state of a gradually adapting process *z_g_*.

$$y_{n}=Iz_{i,n}+(1-I)z_{g,n}$$

The relative weight of the immediate and gradual process is determined by *I*.

The state of the gradual process is updated on every trial, by a certain proportion *G* of the difference between the adaptation state and the target position *u_n_*. This corresponds to the original state-space model by Srimal et al. (2008).

$$z_{g},(n+1)=z_{g,n}-G(z_{g,n}-u_{n})$$

The state of the immediate process is also updated on every trial, but by the full difference between the adaptation state and the target position, so that the state always corresponds to the target position in the previous trial.

$$z_{i},(n+1)=u_{n}$$

To start the model, an initial value of the two states has to be specified. For this purpose, we used the initial gain as the average saccade amplitude in the first 10 trials.

The primary purpose of the model was to distinguish between an immediate adjustment and a gradual adaptation. Hence the model is limited and does not capture spontaneous recovery (Ethier et al., 2008). Please note that the immediate adjustment and the gradual adaptation may not directly map onto the slow and fast adaptation process identified earlier (Ethier et al., 2008). In particular, the immediate-adjustment process may rather be understood as a voluntary selection mechanism, unlike the slow adaptation process.

## Results

### Fixation Baseline

**FIGURE 2:**
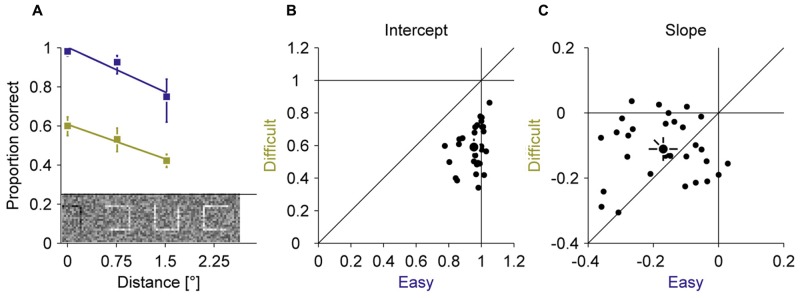
**Experiment 1, perceptual performance during fixation.** Difficult and easy conditions are shown in yellow and blue, respectively. **(A)** Average proportion correct as a function of the squares eccentricity. **(B)** Intercept of the linear fits from **(A)**. **(C)** Slopes of the linear fits from **(A)**. Small dots represent data for individual observers; large dots represent the mean across observers; Error bars represent 95% confidence intervals.

In all experiments, we used a compound of seven squares as stimulus. In a fixation baseline, we first assessed how well observers can discriminate the location of a gap in squares at different positions in the compound when they are fixating the central square (**Figure 2**). Those three different eccentricities spanned the expected range of errors during the adaptation experiment. Two gap sizes (easy 100% and difficult 20%) were used to manipulate the difficulty. By fitting linear regressions of proportion correct on eccentricity, we found that perceptual performance declined with increasing eccentricity for the easy (–0.17 proportion correct over eccentricity ± 0.12) and the difficult (–0.11 proportion correct over eccentricity ± 0.09) condition. This decline was slightly larger for the easy than for the difficult condition [*t*(28) = –2.15; *P* = 0.040]. The overall performance was considerably lower in the difficult (0.59 proportion correct ± 0.13) than the easy (0.95 proportion correct ± 0.07) condition [*t*(28) = 15.03; *P* < 0.001]. These baseline measurements showed that the discrimination task required foveal acuity and that the manipulation of task difficulty by gap size was successful.

### Target Visibility as Possible Error Correction Signal (Experiment 1)

In Experiment 1, we investigated the hypothesis that the effect of a perceptual task on saccade adaptation is mediated by the visibility of the task-relevant object. This hypothesis assumes that the visual system has an internal representation about the stimulus uncertainty (Barthelme and Mamassian, 2009, 2010) and that this uncertainty can be used to optimize eye movement control (Najemnik and Geisler, 2005; Geisler et al., 2006; Najemnik and Geisler, 2008). If this is the case, saccadic adaptation should be weaker and slower if the perceptually relevant object is easy to discriminate and stronger and faster if the perceptually relevant object is difficult to see.

**FIGURE 3:**
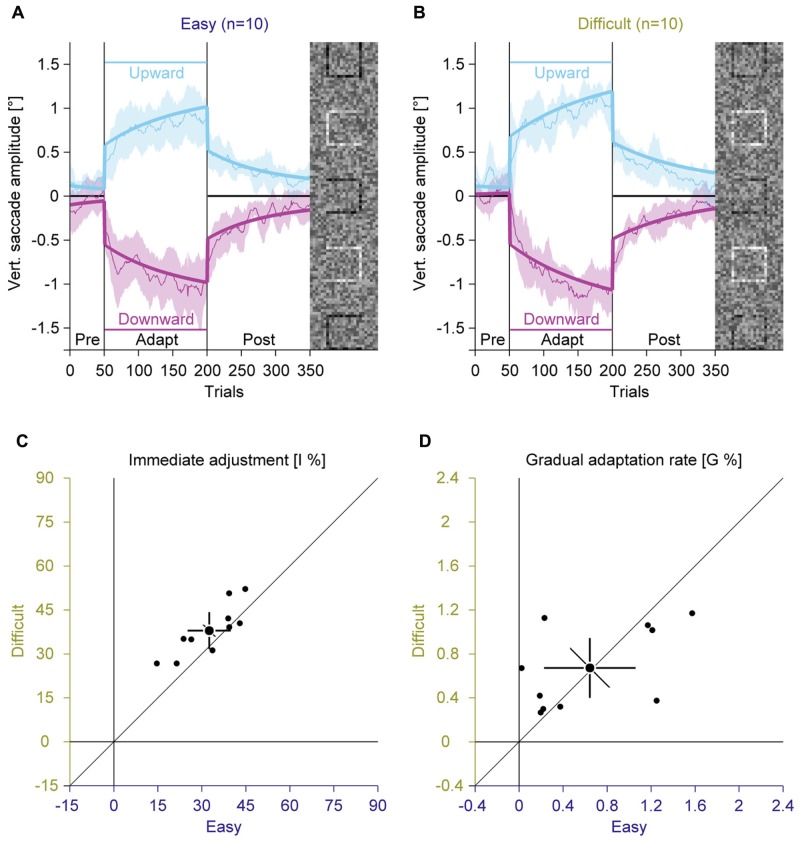
**Experiment 1, saccadic adaptation with easy and difficult perceptual task.** **(A)** Saccadic adaptation with an easy perceptual task. **(B)** Saccadic adaptation with a difficult perceptual task. **(A,B)** The thin lines represent the average across observers. Data are smoothed by a running average with a bin size of 10 trials, for display. The shaded regions represent 95% confidence intervals. The thick lines represent the two-state model fit. Upward and downward adaptation is shown in blue and red, respectively. The vertical lines indicate the onset and offset of the adaptation phase. The horizontal lines indicate the location of the black discrimination square. In each trial, only one of the squares was displayed in black. **(C)** Immediate adjustment in the two-state model fits. **(D)** Gradual adaptation rate in the two-state model fits. **(C,D)** Small dots represent data for individual observers; large dots represent the mean across observers; Error bars represent 95% confidence intervals.

In two separate adaptation conditions, we used the easy and the difficult gap condition to induce saccadic adaptation by presenting the task-relevant square at eccentric locations in the compound stimulus (**Figure 1**). We analyzed the vertical amplitude of the saccades to assess the influence of the perceptual task on the saccade direction (**Figures 3A,B**; Supplementary Figure S1). In the pre-adaptation phase, vertical saccade amplitudes were close to zero in the easy (upward: 0.13° ± 0.10; downward: –0.06° ± 0.26) and the difficult condition (upward: 0.20° ± 0.20; downward: 0.01° ± 0.16). At the end of the adaptation phase, vertical saccade amplitudes differed according to the location of the discrimination square for the easy (upward: 0.87° ± 0.36; downward: –1.03° ± 0.41) and the difficult condition (upward: 1.06° ± 0.24; downward: –1.07° ± 0.31). This effect of adaptation was confirmed by a significant interaction between phase and direction [*F*(1,9) = 200.33, *P* < 0.001]. However, there was no main effect of task difficulty and also no significant interaction with this factor (all *P*s ≥ 0.171). These results indicate that the perceptual task induced reliable saccadic adaptation, but task difficulty had no effect on the overall magnitude of adaptation.

Inspection of the time course of the adaptation revealed that there were two distinct phases of adaptation (**Figures 3A,B**): a rather quick adjustment when the location of the discrimination square changed and a slower, gradual changes in saccade amplitudes afterward. To account for these two components, we used a model that combines an immediate and a gradual error correction process (Schütz et al., 2014). The previous study showed that such a model explains the adaptation data better than single-state models, using an immediate or a gradual error correction alone. In the easy condition the immediate adjustment (**Figure 3C**; 32.52% ± 10.28) was significantly smaller [*t*(9) = –3.01; *P* = 0.015] than in the difficult condition (37.92% ± 8.83). Both values were significantly larger than zero (all *P*s < 0.001) and were highly correlated [*r*(9) = 0.83; *P* = 0.001]. The gradual adaptation (**Figure 3D**) was 0.64% ± 0.58 in the easy condition and 0.67% ± 0.38 in the difficult condition. Both values were significantly larger than zero (all *P*s < 0.007). They did not differ significantly from each other [*t*(9) = –0.18; *P* = 0.860] and tended to be correlated [*r*(9) = 0.52; *P* = 0.060]. These results suggest that task difficulty only affected the immediate adjustment, but not the gradual adaptation of saccade direction.

To further investigate if task difficulty affects adaptation, we correlated intercepts and slopes from the fixation baseline and the average perceptual hit rate in the adaptation experiment with the immediate adjustment and the gradual adaptation rate. None of these indicators of perceptual performance was correlated with any eye movement learning parameter (all *P*s ≥ 0.253). Hence also these more detailed measurements of task difficulty on an individual level did not support the hypothesis that the effect of a perceptual task on saccadic adaptation is mediated by the visibility of the perceptual target. This suggests that the amount of information that can be gained by adapting the saccades did not modulate the rate of adaptation.

We analyzed saccade latencies to test the hypothesis that observers delayed their saccades to improve saccadic control (Supplementary Figure S2). Indeed, we found a main effect of phase for saccade latencies [*F*(1,9) = 8.08, *P* = 0.019], but saccade latencies actually *de*creased from the pre-adaptation (150 ms ± 29) to the end of the adaptation phase (142 ms ± 24). This is inconsistent with the assumption that the observed changes in saccade amplitudes were achieved by delaying the saccades. There were also no statistically significant correlations between saccade latencies or changes in saccade latencies between pre- and adaptation phase with the magnitude of immediate and gradual adaptation. Thus, saccade latencies did not influence the magnitude of adaptation.

### Task Feedback as Possible Reinforcement for Saccades (Experiment 2)

In Experiment 2, we asked whether the feedback about the perceptual judgment acts as a reinforcer driving saccadic adaptation. To test this hypothesis, we measured saccadic adaptation with valid feedback and with random feedback, meaning that feedback was not related to the perceptual judgment.

**FIGURE 4:**
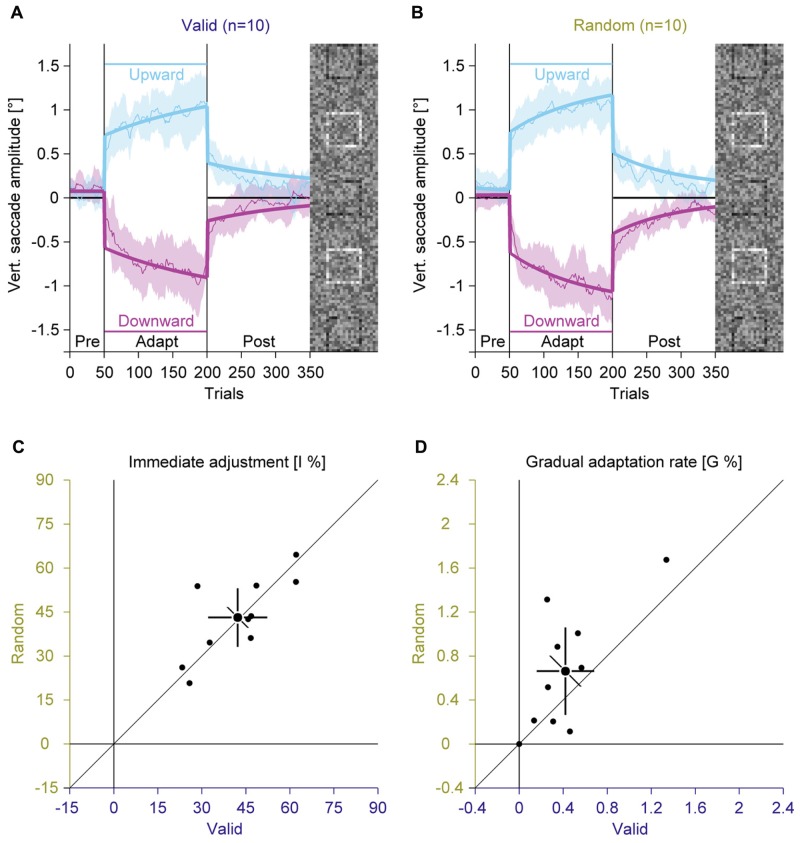
**Experiment 2, saccadic adaptation with valid and random feedback about the perceptual judgement.** **(A)** Saccadic adaptation with valid feedback about the perceptual judgement. **(B)** Saccadic adaptation with random error feedback about the perceptual judgement. **(C)** Immediate adjustment in the two-state model fits. **(D)** Gradual adaptation rate in the two-state model fits. **(A–D)** Conventions are the same as in **Figure 3**.

Like in Experiment 1, we compared average vertical saccade amplitudes in the pre-adaptation phase and at the end of the adaptation to assess the general magnitude of adaptation (**Figures 4A,B**; Supplementary Figure S3). In the pre-adaptation phase, vertical saccade amplitudes were close to zero in the valid (upward: 0.06° ± 0.16; downward: 0.12° ± 0.16) and the random condition (upward: 0.13° ± 0.18; downward: 0.02° ± 0.12). At the end of the adaptation phase, vertical saccade amplitudes differed according to the location of the discrimination square for the valid (upward: 1.02° ± 0.42; downward: –0.86° ± 0.40) and the random condition (upward: 1.08° ± 0.32; downward: –1.04° ± 0.32). This effect of adaptation was confirmed by a significant interaction between phase and direction [*F*(1,9) = 119.62, *P* < 0.001]. However, there was no main effect of feedback and also no significant interaction with this factor (all *P*s ≥ 0.132). These results indicate that the perceptual task induced reliable saccadic adaptation that was not influenced by the feedback about the perceptual judgment.

Like in Experiment 1, we fitted the two-state model. The immediate adjustment (**Figure 4C**) was 42.23% ± 14.11 in the valid and 43.12% ± 13.95 in the random condition, both were significantly larger than zero (all *P*s < 0.001). These values were significantly correlated [*r*(9) = 0.75; *P* = 0.006] and did not differ significantly [*t*(9) = –0.28; *P* = 0.782]. The gradual adaptation (**Figure 4D**) was 0.42% ± 0.37 in the valid and 0.66% ± 0.56 in the random condition, both were significantly larger than zero (all *P*s ≤ = 0.005). There was a significant correlation between conditions [*r*(9) = 0.71; *P* = 0.010], but the gradual adaptation tended to be smaller for the valid than for the random condition [*t*(9) = –1.95; *P* = 0.083]. This trend is opposite to what was expected: that feedback is used as a teaching signal for adaptation. In that case the gradual adaptation should be larger for the valid than the random condition.

In conclusion, the results of Experiment 2 do not show any clear difference between valid and random feedback about the perceptual judgment. Hence the results do not support the hypothesis that reinforcement from the feedback about the perceptual task drives the change in eye movement parameters.

### Effects of Training (Experiments 1 and 2)

**FIGURE 5:**
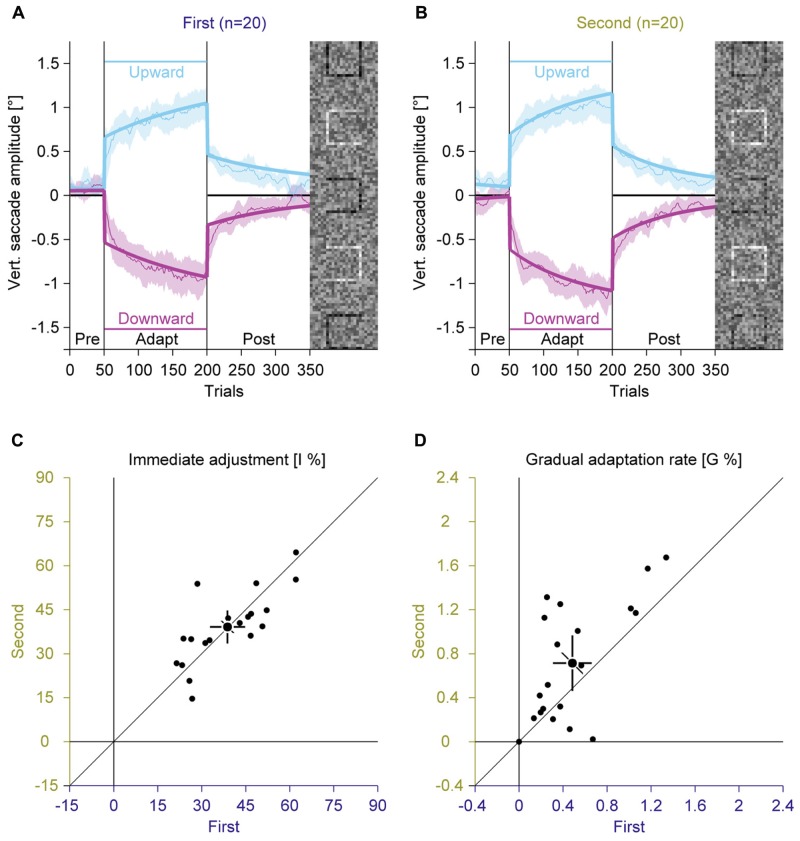
**Experiments 1 and 2, saccadic adaptation for the first and the second session.** **(A)** Saccadic adaptation in the first session. **(B)** Saccadic adaptation in the second session. **(C)** Immediate adjustment in the two-state model fits. **(D)** Gradual adaptation rate in the two-state model fits. **(A–D)** Conventions are the same as in **Figure 3**.

Experiment 2 showed a trend for a stronger gradual adaptation with random feedback than with valid feedback (**Figure 4D**). Since the valid feedback condition was always recorded first, it might be that this trend reflects a facilitation of re-adaptation, called savings (saccades: Kojima et al., 2004; hand movements: Krakauer et al., 2005), rather than an effect of the type of feedback. To test this hypothesis, we joined the data from Experiments 1 and 2 and sorted conditions according to the order of recording (**Figures 5A,B**).

In the pre-adaptation phase, vertical saccade amplitudes were close to zero in the first (upward: 0.09° ± 0.14; downward: 0.03° ± 0.23) and the second session (upward: 0.17° ± 0.19; downward: 0.02° ± 0.14). At the end of the adaptation phase, vertical saccade amplitudes differed according to the location of the discrimination square for the first (upward: 0.94° ± 0.39; downward: –0.95° ± 0.40) and the second session (upward: 1.07° ± 0.28; downward: –1.05° ± 0.31). This effect of adaptation was confirmed by a significant interaction between phase and direction [*F*(1,19) = 302.15, *P* < 0.001]. There was a trend for an interaction between session and direction [*F*(1,19) = 3.63, *P* = 0.072], but the main effect of session and the other interactions were clearly not significant (all *P*s ≥ 0.159).

Like in Experiment 1, the two-state model revealed a dissociation between immediate and gradual error correction processes. The immediate adjustment (**Figure 5C**) was 38.78% ± 12.84 in the first and 39.11% ± 12.08 in the second session, both were significantly larger than zero (all *P*s < 0.001). These values were significantly correlated across sessions [*r*(9) = 0.75; *P* < 0.001] and did not differ significantly from each other [*t*(19) = –0.17; *P* = 0.869]. The gradual adaptation (**Figure 5D**) was with 0.49% ± 0.38 significantly smaller in the first than in the second session with 0.71% ± 0.54 [*t*(19) = –2.50; *P* = 0.022]. Both values were significantly larger than zero (all *P*s < 0.001) and significantly correlated [*r*(9) = 0.65; *P* = 0.001]. These results indicate that the gradual adaptation rate, but not the immediate adjustment improved from training from the first to the second session. Hence the trend for a larger gradual adaptation with random feedback in Experiment 2 might reflect savings because this condition was recorded second.

### Target Selection vs. Perceptual Task (Experiment 3)

**FIGURE 6:**
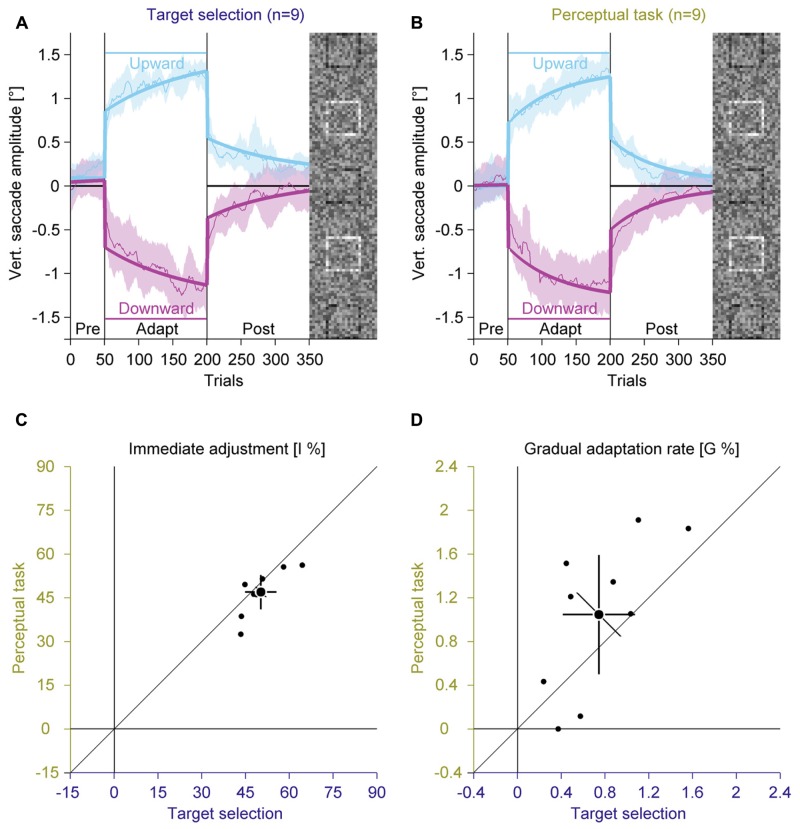
**Experiment 3, saccadic adaptation with instruction for target selection and with perceptual task.** **(A)** Saccadic adaptation with instruction for target selection. **(B)** Saccadic adaptation with perceptual task. **(C)** Immediate adjustment in the two-state model fits. **(D)** Gradual adaptation rate in the two-state model fits. **(A–D)** Conventions are the same as in **Figure 3**.

Experiments 1 and 2 showed that target visibility and feedback about the perceptual task have only negligible effects on adaptation. Hence it might be that the perceptual task merely triggers a target selection mechanism, which then drives saccadic adaptation. To test this hypothesis, we compared adaptation in a condition with a simple targeting instruction and adaptation in a condition with the difficult perceptual task (**Figures 6A,B**; Supplementary Figure S4).

In the pre-adaptation phase, vertical saccade amplitudes were close to zero in the target selection (upward: 0.17° ± 0.13; downward: 0.06° ± 0.24) and in the perceptual task condition (upward: 0.04° ± 0.25; downward: 0.05° ± 0.28). At the end of the adaptation phase, vertical saccade amplitudes differed according to the location of the target square in the target selection condition (upward: 1.25° ± 0.16; downward: –1.16° ± 0.32) and in the perceptual task condition (upward: 1.26° ± 0.25; downward: –1.13° ± 0.39). This effect of adaptation was confirmed by a significant interaction between phase and direction [*F*(1,8) = 580.71, *P* < 0.001]. However, there was no main effect of instruction and also no significant interaction with this factor (all *P*s ≥ 0.117).

Like in the previous experiments, we fitted the two-component model. The immediate adjustment (**Figure 6C**) was 50.25% ± 7.00 in the target selection condition and 46.94% ± 7.63 in the perceptual task condition, both were significantly larger than zero (all *P*s < 0.001). These values correlated significantly [*r*(8) = 0.80, *P* = 0.004], but the immediate adjustment tended to be smaller for the perceptual task than for target selection condition [*t*(8) = 2.15; *P* = 0.064]. The gradual adaptation (**Figure 6D**) was 0.74% ± 0.43 in the target selection and 1.05% ± 0.71 in the perceptual task, both were significantly larger than zero (all *P*s ≤ = 0.002). These values correlated significantly [*r*(8) = 0.68, *P* = 0.021] and did not differ significantly from each other [*t*(8) = –1.74; *P* = 0.119]. These results indicate that an instruction for target selection had similar effects as the perceptual task.

### Target Selection vs. Bottom-Up Visual Effects (Experiment 4)

**FIGURE 7:**
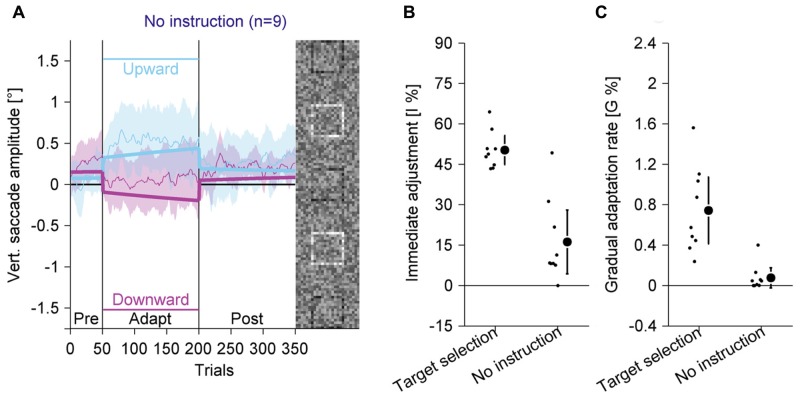
**Experiment 4, saccadic behavior with identical stimuli as in Experiment 3, but without instruction for target selection and without perceptual task.** **(A)** Saccadic behavior without instruction for target selection. **(B)** Immediate adjustment in the two-state model fits. **(C)** Gradual adaptation rate in the two-state model fits. **(B,C)** Data from the target selection condition from Experiment 3 are replotted. **(A–C)** Conventions are the same as in **Figure 3**.

To rule out the possibility that the adaptation effects in the previous experiments were merely triggered by the visual salience of the black square, we ran an experiment in which observers were instructed that they should saccade to the peripheral compound and that the individual squares of the compound and their colors were irrelevant to the task (**Figure 7A**; Supplementary Figure S5). We compared the results between-subjects to the results of the target-selection condition of Experiment 3.

In the pre-adaptation phase, vertical saccade amplitudes were close to zero (upward: 0.07° ± 0.27; downward: 0.26° ± 0.31). At the end of the adaptation phase, vertical saccade amplitudes were still close to zero (upward: 0.50° ± 0.56; downward: 0.09° ± 0.47) and there was no statistically significant interaction between phase and direction [*F*(1,8) = 3.14, *P* = 0.114]. Most importantly, when the data from the target selection condition in Experiment 3 were included, there was a significant three-way interaction of phase, direction and the between-subjects factor condition [*F*(1,16) = 23.55, *P* < 0.001]. The difference between upward and downward directions at the end of the adaptation phase was almost six times larger with than without target selection instruction. Hence, the adaptation effects were significantly weaker if observers were instructed to look at the peripheral compound than when they were instructed to look at the black square.

We also analyzed the two-state model fits. The immediate adjustment (**Figure 7B**) was 16.21% ± 15.39, significantly larger than zero [*t*(8) = 3.16; *P* = 0.013] but significantly smaller than in the target selection condition [*t*(16) = 6.04; *P* < 0.001]. The gradual adaptation (**Figure 7C**) was 0.08% ± 0.13, not significantly different from zero [*t*(8) = 1.80; *P* = 0.109] and significantly smaller than in the target selection condition [*t*(16) = 4.46; *P* < 0.001]. These results show that the small changes in vertical saccade amplitude were mostly due to immediate adjustments when the black square changed its location and that there was no gradual adaptation. This confirms previous findings (Schütz et al., 2014) and shows that adaptation in the other experiments was not caused by bottom-up visual signals from the black square, but by top-down signals identifying the black square as the task-relevant element or as the eye movement target.

### Learning Components across Experiments (Experiments 1 to 3)

To further differentiate between immediate and gradual error correction, we compared the parameters of the two-state model in Experiments 1 to 3. While there were strong inter-individual differences in the magnitude of the immediate adjustment (ranging from 14.63 to 64.51%, applied only once when the task-relevant location changes) and the gradual adaptation (ranging from 0 to 1.91%, applied in every trial) across experiments, these differences were quite stable, since there were large correlations between repeated measurements for the immediate adjustment [*r*(28) = 0.79, *P* < 0.001] and the gradual adaptation [*r*(28) = 0.60, *P* < 0.001]. Interestingly, the magnitude of immediate adjustments and gradual adaptation was not correlated [*r*(28) = 0.04, *P* = 0.823], which supports the assumption that these values represent two dissociable mechanisms. This notion is also supported by the findings that task difficulty only modulated the immediate adjustment (**Figure 3**) while repetition only modulated the gradual adaptation (**Figure 5**).

## Discussion

In this study, we investigated how a perceptual task induces saccadic adaptation (Schütz et al., 2014). In this paradigm adaptation is induced by placing a task-relevant black square at different locations within a larger peripheral compound. In four experiments, we compared the magnitude of adaptation with an easy and a difficult perceptual task (Experiment 1, **Figure 3**), with valid or random feedback about the perceptual judgment (Experiment 2, **Figure 4**) and with a perceptual task or an instruction either to merely fixate the black square (Experiment 3, **Figure 6**) or to look to the peripheral compound (Experiment 4, **Figure 7**). In all experiments but the last one, we found robust saccadic adaptation, with only small differences between conditions. The last experiment was the only experiment that did not require a top-down signal to aim toward the black square. This is further evidence that saccadic adaptation can be induced by a top-down signal, even in the absence of a prediction error and a bottom-up retinal position error.

Several findings support the assumption that these adaptation effects are genuine modifications of the sensorimotor-transformation for saccadic eye movements. First, there are clear post-adaptation effects, in the sense that the newly acquired saccade metrics have to be unlearned when the task-relevant element is displayed again at the central location. Second, a previous study showed that the adaptation with the perceptual task transfers at least to some degree to saccades that are not task related (Schütz et al., 2014). Since this paradigm does not require any gaze-contingent manipulations like in the intra-saccadic step paradigm (McLaughlin, 1967), it could prove to be useful to study adaptation when online readings of eye movements or gaze contingent display changes are not available, such as in clinical settings.

### Potential Mediators of Perceptual Task Induced Adaptation

Since eye movements in visual search maximize the information gain (Najemnik and Geisler, 2005, 2008; Geisler et al., 2006), we hypothesized that adaptation in our paradigm is modulated by the amount of information that can be gained by the adaptation of eye movements. Thus we compared adaptation with a difficult and an easy perceptual task in Experiment 1 (**Figure 3**). In favor of the hypothesis, the immediate adjustment was larger in the difficult than in the easy condition. However, the gradual adaptation rate did not differ between difficult and easy conditions. This is evidence for a dissociation between immediate and gradual processes in our paradigm, adding to the finding that the magnitude of those components was not correlated. Presumably the immediate adjustment represents a voluntary, strategic process. Since, humans have access to a representation of visual uncertainty (Barthelme and Mamassian, 2009, 2010), it is possible that observers voluntarily shifted their saccades toward the perceptual target more in the difficult condition. The gradual process however might represent a more automatic mechanism for reducing the distance between saccade endpoints and task goals, similar to saccade adaptation in intra-saccadic step paradigms (Srimal et al., 2008).

Since, saccadic adaptation can be driven by visual or auditory reinforcement signals (Madelain et al., 2011), we hypothesized that feedback about the perceptual judgment might be inducing saccadic adaptation in our paradigm. We did not find any evidence supporting this hypothesis. First, observers showed robust adaptation in Experiment 1, although they did not receive any feedback about their judgments. Second, false feedback in Experiment 2 did not hamper adaptation in any way (**Figure 4**). Thus, we conclude that the effect of a perceptual task on saccadic eye movements is not mediated by external feedback about the perceptual task. We must note that although it is clear from our results that feedback does not mediate the perceptual task adaptation effect, we cannot definitively claim that it has no modulatory role in the expected direction. Indeed, we found an effect of order—i.e., faster gradual adaptation on the second session—that could have masked a deterioration of performance in the random feedback condition, since it was run after the valid feedback condition. However, there are two theoretical reasons why feedback about the perceptual judgment might be totally ineffective. First, external feedback is a very indirect reinforcement for eye movements because the accuracy of the perceptual judgment does not only depend on the accuracy of eye movements. Internal sensory noise as well as sampling efficiency fluctuates across trials impeding the correlation between eye movements and perception, especially at threshold. Second, feedback about the accuracy of the perceptual judgment might be informative about whether an eye movement was useful or not, but it does not provide information about how to optimize the eye movement. However, a previous study could induce adaptation with simple reinforcement that did not contain directional information (Madelain et al., 2011).

As target visibility and task feedback did not affect adaptation much and since the adaptation was similar with perceptual task and with an instruction to fixate the black square, it is likely that a perceptual task triggers adaptation by a target selection mechanism. Previous studies showed nicely that in classical intra-saccadic step paradigms, adaptation is selective to the saccade target and not affected by the location of a distractor object (Madelain et al., 2010) or the scene background (Madelain et al., 2013). We can think that the perceptual task defines the discrimination square as eye movement target. Because of the global effect, saccades are directed to the center of gravity of a compound stimulus (Findlay, 1982; for review, see Van der Stigchel and Nijboer, 2011), which can generate a post-saccadic error relative to the task-defined eye movement target. Over several trials this top-down defined error is corrected. An alternative strategy to improve saccade accuracy is to increase saccade latencies (van Zoest et al., 2004; Schütz et al., 2012), since the global effect is weaker at longer saccade latencies (Ottes et al., 1985). However, our observers did not use this strategy, because saccade latencies were decreased rather than increased across trials, replicating our previous findings (Schütz et al., 2014).

Since there is no or very weak adaptation with the same stimuli when observers are instructed to look at the peripheral compound as opposed to select the black square within the compound (Experiment 4 and Schütz et al., 2014), we can ascertain that the error is not generated by bottom-up salience signals from the different elements in the compound. This experimental paradigm shows that top-down signals not only determine target selection of eye movements (for review, see Schütz et al., 2011), but also the short-term modification of eye movement metrics. More precisley, it suggests that a mismatch between a location selected for perception and the actual saccade landing location can drive adaptation. This correction of a top-down error exemplifies the flexibility of learning in the eye movement system, since other error signals that have been shown to be effective in driving adaptation, such as prediction errors concerning the actual saccade landing location relative to the planned saccade landing location (Bahcall and Kowler, 2000; Wong and Shelhamer, 2011; Collins and Wallman, 2012) are not functional in this paradigm. In our paradigm, predictions based on an efference copy signal should be accurate, because no element in the stimulus changes location during the saccade.

### Dissociation between Immediate and Gradual Error Correction

Using a two-state model, we subdivided the changes in saccade behavior into an immediate adjustment and a gradual adaptation. The results showed that these two states could be clearly identified and dissociated from each other. While either parameter was highly correlated across different experimental conditions, there was no correlation between immediate and gradual changes within one experimental condition. There were also dissociations between immediate and gradual changes with respect to different experimental manipulations. While the immediate adjustment was sensitive to the difficulty of the task (Experiment 1), the gradual adaptation showed savings (Experiments 1 and 2), i.e., a facilitation during re-adaptation (Kojima et al., 2004; Krakauer et al., 2005).

We can ask why there were adaptation effects at all and why observers were not able to direct their saccades to the task-relevant element immediately without learning. We can rule out uncertainty about the *location* of the task-relevant element, because it was clearly distinct from the distractors and because we instructed observers that it will keep its location throughout a block of 100 trials. Furthermore, if the limiting factor was uncertainty about the location of the task-relevant element, one would expect much larger effects in the second run of the experiment, because observers already experienced all possible locations once. However, training effects were small and were only present in the gradual adaptation component, consistent with the effects of repetition on classical saccade adaptation (Kojima et al., 2004). Another possibility is that the transformation from a visual to a motor vector can be modified voluntarily only up to certain limit. The immediate adjustment might represent a more voluntary component of saccade control and its magnitude might reflect this upper limit of modification.

The distinction between an immediate and a gradual error correction process is only meaningful in paradigms in which observers are able to perceive the change in target location between trials. This is typically not the case in the intra-saccadic step paradigm (McLaughlin, 1967), because the step is masked by saccadic suppression of displacement (Bridgeman et al., 1975). Previous research in the intra-saccadic step paradigm distinguished between a slow and a fast adaptation process (Ethier et al., 2008), that differ in learning and retention rates. Since we did not measure retention here, we cannot distinguish between the slow and the fast adaptation process. Presumably the gradual adaptation in our model is a mixture of the slow and the fast adaptation process found by Ethier et al. (2008).

We conjecture that adaptation induced by a perceptual task or target selection might share neural substrates with classical saccadic adaptation. This might be more the case for the gradual error correction than for the voluntary correction mechanism, possibly involving frontal areas. Classical saccadic adaptation has been shown to rely on different neural structures like the cerebellum (Catz et al., 2008; Soetedjo et al., 2008), frontal (Blurton et al., 2012) and parietal cortical areas (Gerardin et al., 2012; Panouilleres et al., 2014), which could be related to separate aspects of oculomotor learning such as target selection, prediction of sensory input and the production of error signals.

## Conclusion

Based on these results we argue that saccadic adaptation is a general mechanism to bring eye movements closer to the intended eye movement target. It might not matter if the eye movement target is defined by bottom-up signals, such as a single object appearing in the periphery or by top-down signals, such as provided by task demands or instruction. It also might not matter if the post-saccadic error is created by an intra-saccadic target step or by inaccurate eye movements, for instance due to averaging of spatially extended targets. Depending on the context, different error signals, such as prediction errors or top-down errors can be used to drive saccadic adaptation.

## Conflict of Interest Statement

The authors declare that the research was conducted in the absence of any commercial or financial relationships that could be construed as a potential conflict of interest.
