# Supplementary material for: Perceptual task induces saccadic adaptation by target selection
Source: Front Hum Neurosci. 2015 Oct 20;9:566. doi: 10.3389/fnhum.2015.00566 (PMC4611985; doi:10.3389/fnhum.2015.00566)
Supplement: Supplementary file 1 [file Data_Sheet_1.PDF]

Supplementary Material

Perceptual task induces saccadic adaptation by target selection

Alexander C. Schütz[1,2]*, David Souto[1,3]

[1] Abteilung Allgemeine Psychologie, Justus-Liebig-Universität Gießen, Gießen, Germany
[2] Allgemeine und Biologische Psychologie, Philipps-Universität Marburg, Marburg, Germany
[3] Department of Neuroscience, Psychology and Behaviour, University of Leicester, Leicester, UK

***Correspondence:** Alexander C. Schütz, alexander.c.schuetz@psychol.uni-giessen.de

1. Supplementary Figures and Tables

1.1. Supplementary Figures

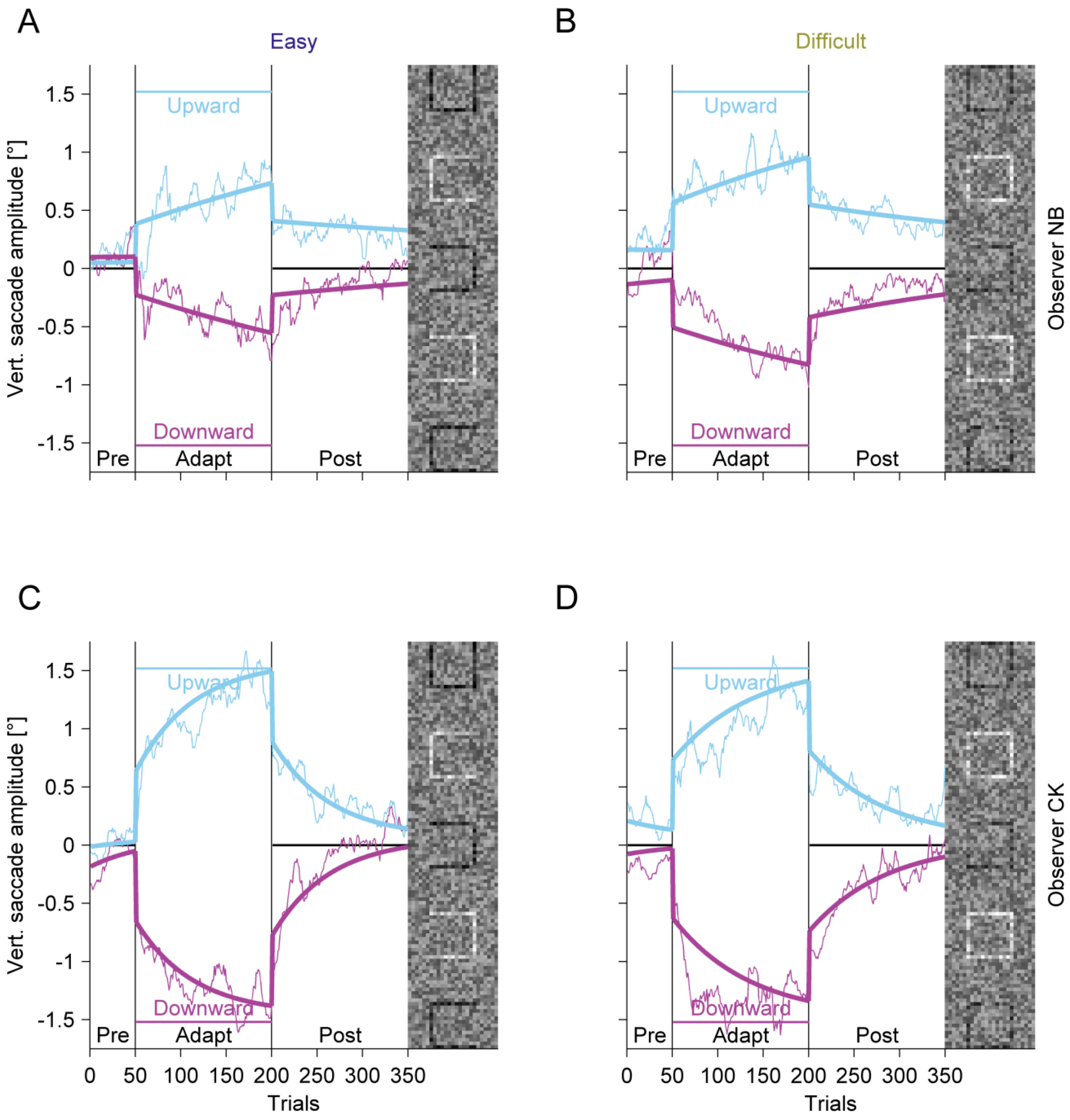

Supplementary Figure 1 | Representative observers in Experiment 1. (A, C) Easy perceptual task. **(B, D)** Difficult perceptual task. **(A, B)** Observer NB. **(C, D)** Observer CK. Conventions are the same as in Figure 3A & B.

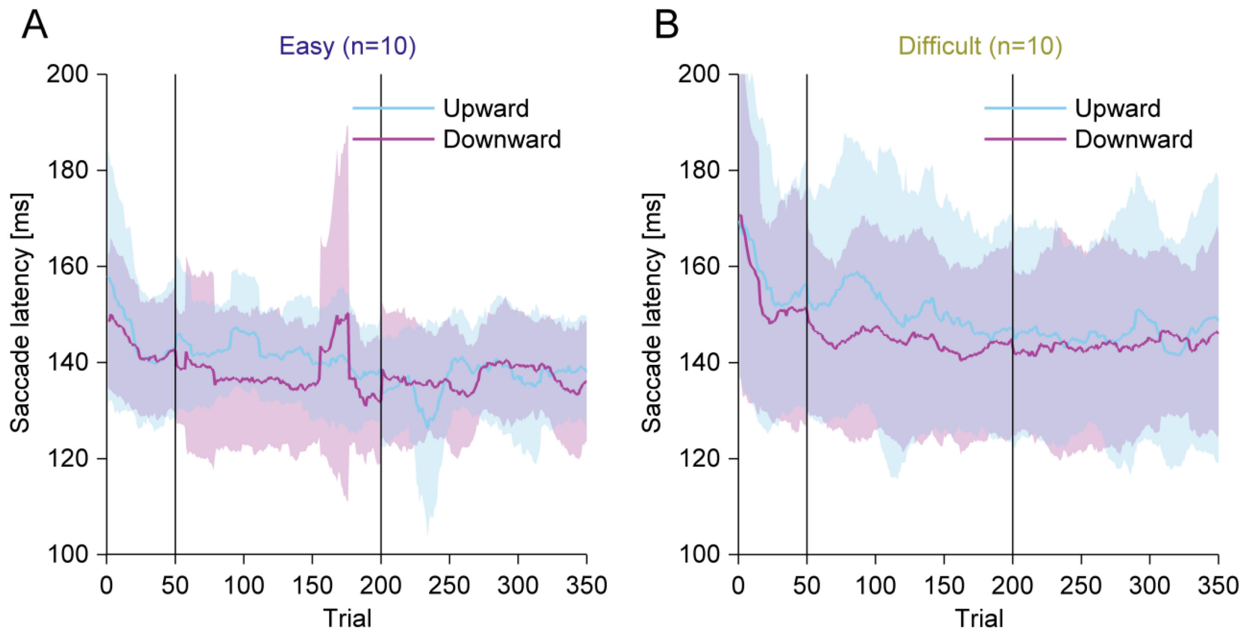

Supplementary Figure 2 | Saccade latencies in Experiment 1. (A) Saccade latencies with an easy perceptual task. **(B)** Saccade latencies with a difficult perceptual task. **(A, B)** The thin lines represent the average across observers. Data are smoothed by a running average with a bin size of 20 trials. The shaded regions represent 95% confidence intervals. Upward and downward adaptation are shown in blue and red, respectively. The vertical lines indicate the onset and offset of the adaptation phase.

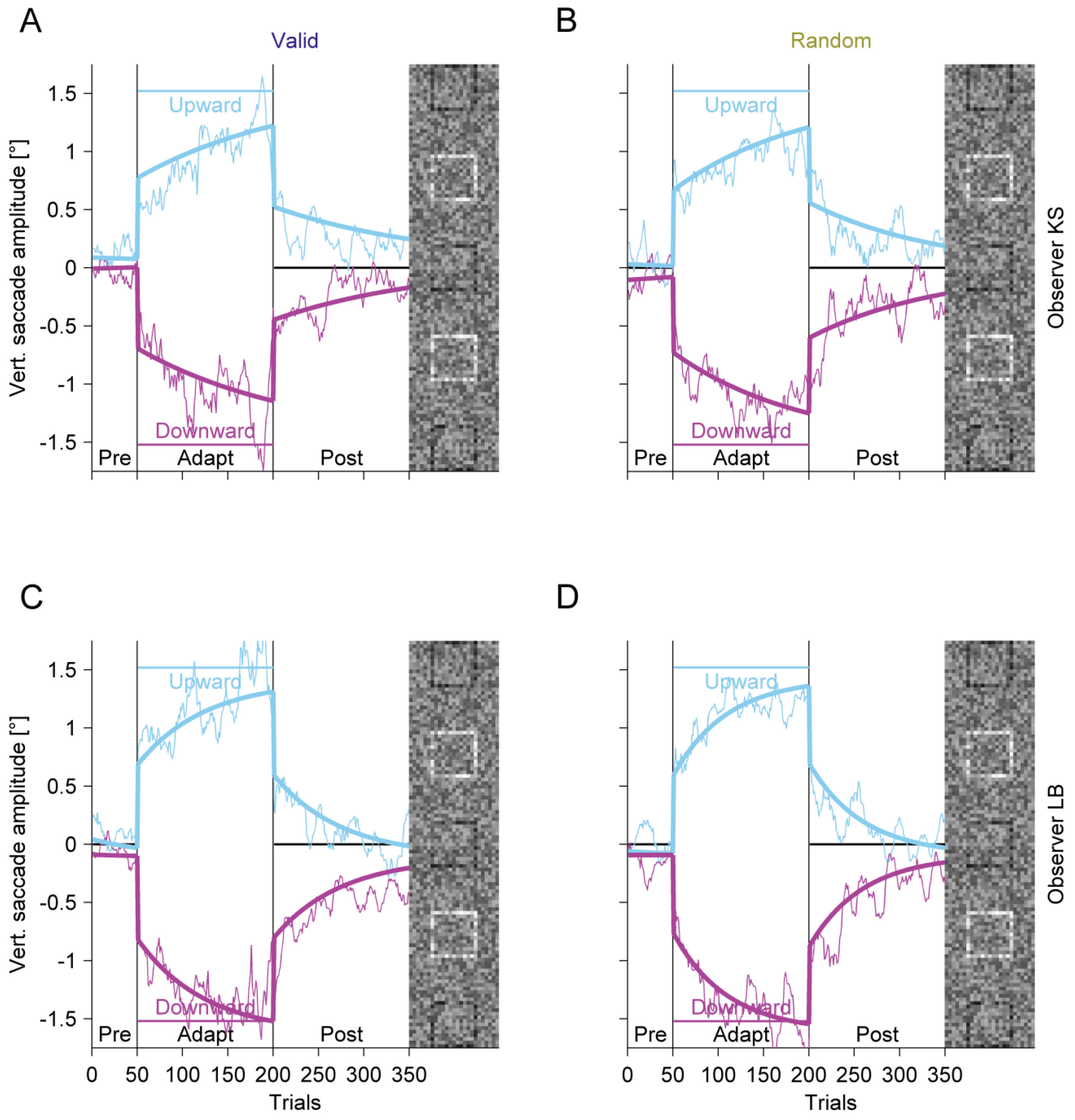

Supplementary Figure 3 | Representative observers in Experiment 2. (A, C) Valid feedback. **(B, D)** Random feedback. **(A, B)** Observer KS. **(C, D)** Observer LB. Conventions are the same as in Figure 3A & B.

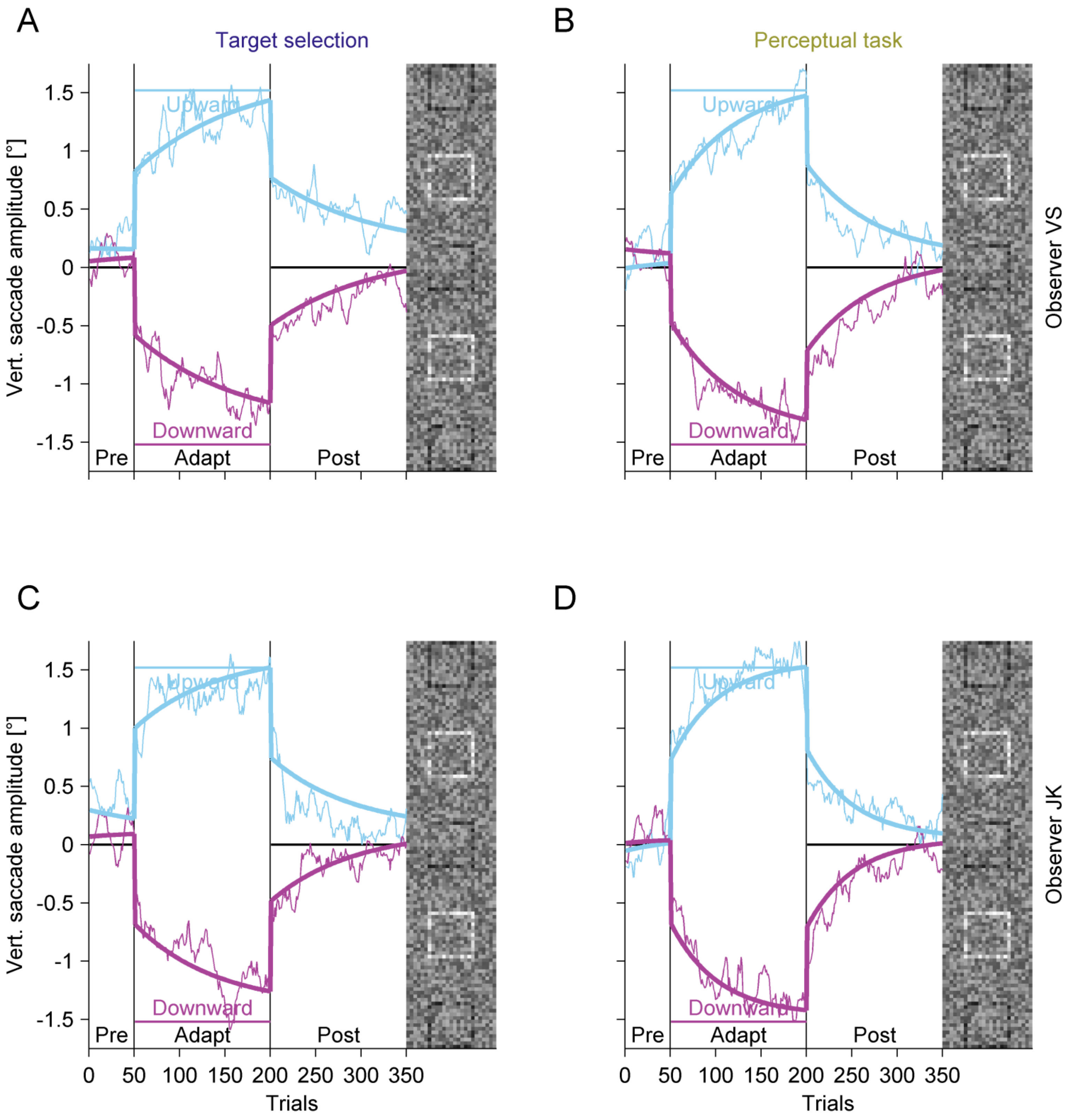

Supplementary Figure 4 | Representative observers in Experiment 3. (A, C) Instruction for target selection. **(B, D)** Perceptual task. **(A, B)** Observer VS. **(C, D)** Observer JK. Conventions are the same as in Figure 3A & B.

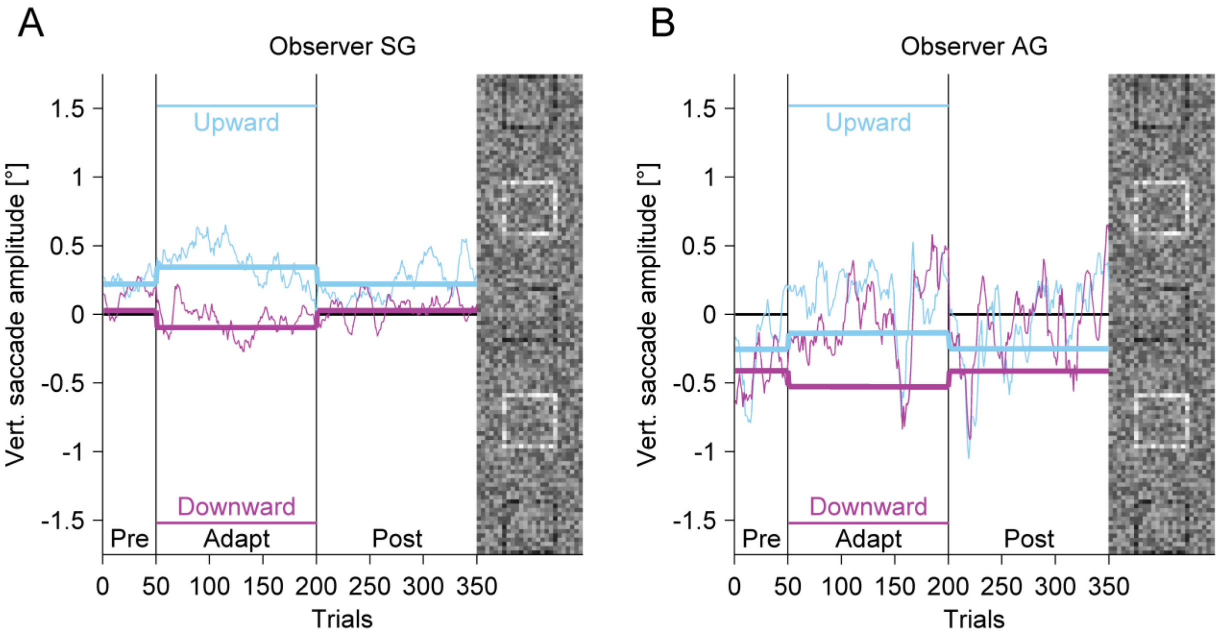

Supplementary Figure 5 | Representative observers in Experiment 4. **(A)** Observer SG. **(B)** Observer AG. Conventions are the same as in Figure 3A & B.
